# Training program for female community volunteers to combat COVID 19 in rural Nepal

**DOI:** 10.1080/16549716.2022.2134425

**Published:** 2022-11-12

**Authors:** Ramu Kharel, Soniya P. Regmi, Timmy Lin, Adam C. Levine, Adam R. Aluisio

**Affiliations:** a Department of Emergency Medicine, Brown University Alpert Medical School, Providence, RI, USA; b Dhulikhel Hospital, Kathmandu University School of Medical Sciences, Dhulikhel, Nepal

**Keywords:** Education, rural Nepal, international education, community education, COVID 19

## Abstract

Female Community Health Volunteers (FCHV) in Nepal have identified lack of appropriate training as a barrier to involvement in the COVID 19 response. With more than 50,000 FCHVs serving rural areas of Nepal, they are instrumental in healthcare and are a major source of information delivery to those with the most limited health-care access in Nepal. This communication describes an innovative training programme to rapidly equip FCHVs with knowledge on COVID 19 response. The ongoing programme leverages partnerships between local municipalities and a local community-based organisation and has rapidly trained more than 300 FCHVs across four districts with a population of 1,000,000, and has plans to expand the training across the country. This training programme is a key example of how local partnerships can be utilised for digital training of FCHVs in remote parts of Nepal and leveraged to strengthen response capacity during the pandemic.

## Introduction

Shortly after the first case of Coronavirus Disease 2019 (COVID 19) was seen in Nepal during January of 2020, the virus reached a wide community spread within a few months [1,2]. The majority of those infected with severe acute respiratory syndrome coronavirus 2 (SARS-CoV-2) can be safely taken care of at home [3]. In Nepal, nearly 97% of those infected were in home isolation [2]. As in the Delta wave, the drastic increase in the number of cases strained the Nepali health system during Omicron wave [4,5]. Though challenges in Nepal are multifactorial, they are largely driven by insufficient healthcare resources, especially in rural areas, which is compounded by limited information and misinformation on the pandemic [6–8].

Female Community Health Volunteers (FCHVs) are an instrumental part of healthcare and are a major source of health information delivery across rural Nepal. Generally, FCHVs are married women locally choses to undergo 18 days of basic training in family planning, maternal/newborn/child health, and nutrition [9]. There are more than 50,000 FCHVs across Nepal. The FCHVs have been key contributors to meeting Millennium Development Goals and other health targets in the country [10]. A FCHV survey conducted after the Nepal earthquake in 2015 found that 100% of those surveyed helped with acute earthquake response, and 94% believed further training would have been beneficial in their response abilities [11].

**Figure 1.**
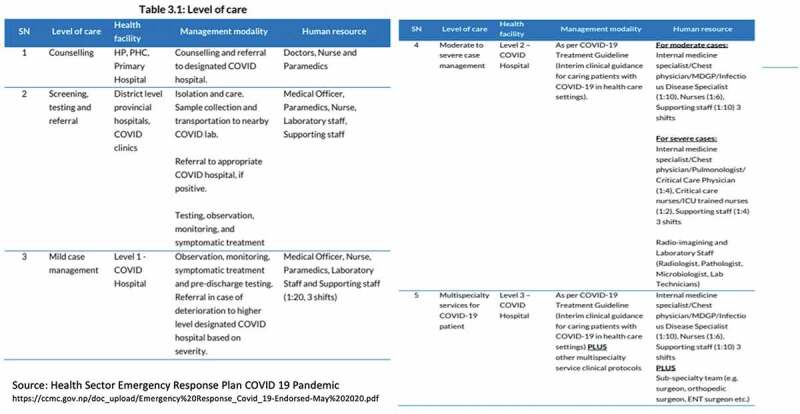
Health sector emergency response plan for different level of care. Nepal Female Community Health Workers’ COVID 19 training.

Though FCHVs have been recognised by the Nepal Ministry of Health and Population (MOHP) as part of COVID 19 Prevention group (CPG); they do not feature prominently in the government care matrix (Figure 1) [12–14]. They are notably missing in the areas where they have proved to be previously impactful in public health actions such as screening, testing, referral, and counseling [13].

**Figure 2.**
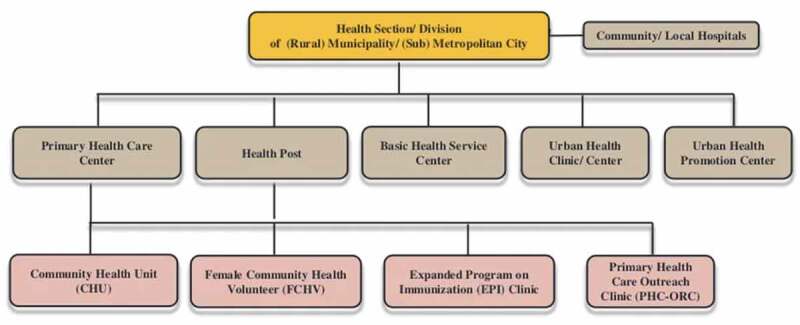
Structure of Nepal’s health sector the level of Municipality/Metropolitan City Level.

Source: https://www.mohp.gov.np/eng/index.php/organogram. Nepal Female Community Health Workers’ COVID 19 Training.

Nepal’s population is approximately 80% rural, and local health posts use FCHVs as the primary mechanism for disseminating health information and awareness in these areas [15]. FCHVs are chosen locally and are the bridge between the rural population and the local health care suppliers (health posts or primary health centres) (Figure 2). Due to their familiarities with local communities, FCHVs are well suited to be community liaisons in COVID 19 education and awareness, and particularly in disseminating accurate data to prevent propagation of misinformation [10,12,16]. A recent survey after the wave caused by the Delta variant in Nepal showed that FCHVs participation in local COVID 19 mitigation efforts was limited due to lack of training and resources (*R. Kharel, personal communication, 28 January 2022*). Community health workers like the FCHVs can be highly effective in pandemic settings, but conditions must be right to facilitate their success [10]. Prior research has shown that FCHVs who receive regular training and support from local health posts were more effective in maternal and child health programmes compared to FCHVs who did not receive appropriate training or support [12].

## Training design and methodology

### Participant selection

To train FCHVs and leverage their penetration to rural areas in the COVID 19 outbreak, Health Advancement Program to Serve All (HAPSA) Nepal®, a Nepali grassroots organisation, partnered with municipalities and other local non-governmental organisations (NGOs) across the country to rapidly deploy a training course aimed at building the capacity of FCHVs in pandemic response. HAPSA Nepal had build partnership with municipal governments in its prior work supporting COVID-19 response (more specifically, designing and distributing home isolation kits), and these pre-existing relationships were leveraged to form partnership with local government or other local NGOs in order to facilitate the implementation of the training.

### Training programme

A three-module virtual training programme was piloted to build the knowledge of FCHVs in the competency areas and was adapted from a training developed by Project HOPE®, an international humanitarian organisation, and faculty from Brown University with specialisations in infectious diseases, emergency care, humanitarian response, and education, to fit the Nepali context [17]. The training is based on vetted resources which were updated prior to each training session.

**Figure 3.**
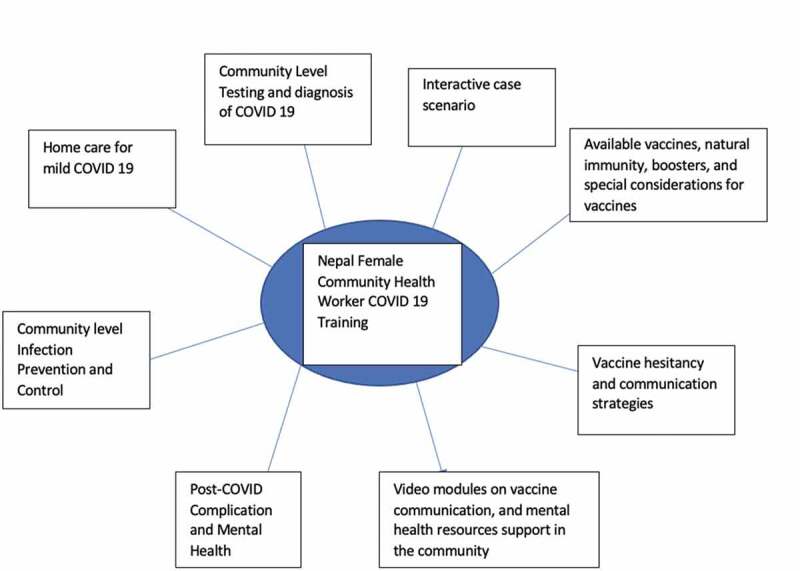
FCHV COVID 19 training topical areas. Nepal Female Community Health Workers’ COVID 19 Training.

The training was designed to provide practical knowledge to allow FCHVs to help in local community response against COVID 19. The training components are illustrated in Figure 3 which include a wide range of key topics pertaining to the pandemic. Training programme includes interactive didactic modules, discussions, case-based learning, and video simulations, and is delivered over a four-hour period including pre-test and post-test assessments.

The training was translated in Nepali, and accuracy and context understanding were checked with back translation. The programme was delivered by a lead facilitator fluent in Nepali with health-care training with logistical support HAPSA Nepal®, and other local partners. The first training was conducted in person, whereas the remaining trainings were conducted virtually.

### Current progress

**Figure 4.**
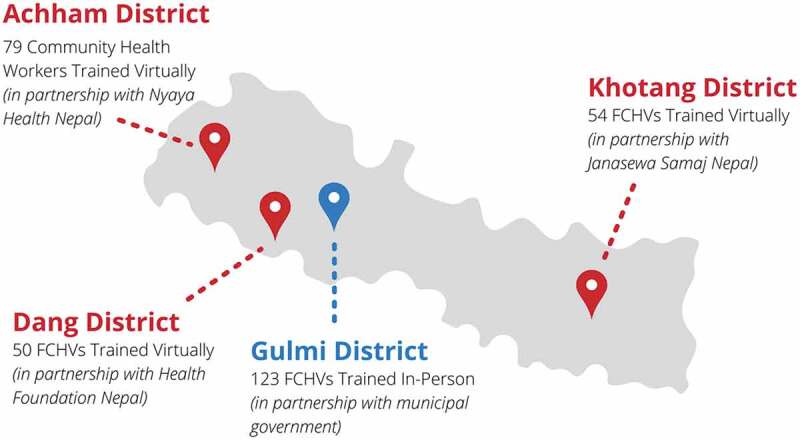
Trainings across Nepal as of March 7th, 2022. Nepal Female Community Health Workers’ COVID 19 Training.

The training programme started during the rise of the Omicron variant in Nepal, and thus far in implementation, has been conducted in four different districts of Nepal: Dang, Gulmi Achham, and Khotang. The cumulative total population covering these districts is more than 1 million. Gulmi’s training was conducted in-person, whereas Dang, Achham and Khotang training were conducted virtually. The in-person training was delivered to 123 FCHVs (which accounted for all FCHVs registered in the Musikot municipality in Gulmi), and subsequent virtual trainings have included 50 FCHVs from Dang, 79 Community Health Workers from Achham, and 54 FCHVs in Khotang. In Gulmi, the local government (municipal government) was the primary coordinator of the training programme. The municipal health administrator was the point of contact, and the mayor and vice-mayor were present for the entire training as well. Subsequent training was conducted virtually in Dang, Achham and Khotang in partnership with local NGOs which led the coordination of training (Figure 4). HAPSA Nepal® and these NGOs and municipalities have a prior working relationship which allowed for this training to be conducted in a timely fashion.

### Future trainings

Active partnerships are being formed with municipalities and other NGOs to disseminate the training across other parts of the country. The recording from the first virtual training has been released online in open access format and has been added to the COVID 19 digital learning [18,19].

### Knowledge acquisition assessment

**Figure 5.**
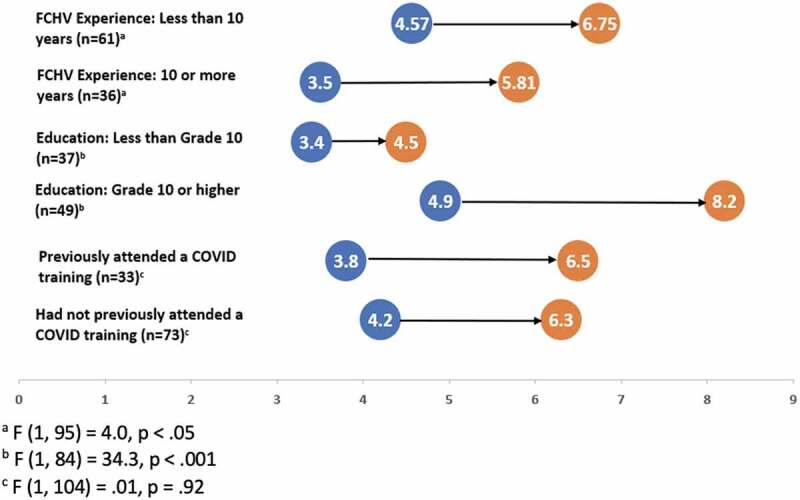
COVID knowledge change from pre to post by FCHV experience, educational attainment, and prior training experience FCHVs from Musikot (*n *= 106). Nepal Female Community Health Workers’ COVID 19 Training.

A 13-question pre- and post-test assessment was conducted, and paired-t test was used to assess knowledge gain. Preliminary results from the in-person training show a statistically significant increase in overall mean knowledge scores from 4.1 to 6.3 (t (105) = 7.8, *p* < 0.001). Stratified analysis was conducted using repeated measure analysis of variance to assess the impact of prior training, FCHV education level, and years of work experience. Repeated measure analysis of variance was conducted to assess the change in knowledge from pre- to post-test across different stratification. Knowledge gain was significantly higher among those with less than 10 years of FCHV experience than those with 10 or more years (F (1, 95) = 4.0, *p* < .05). Knowledge gain was also higher among those with educational level 10th or higher compared with less than 10th grade educational level (F (1, 84) = 34.3, *p* < .001). There was no statistically significant difference in knowledge when comparing those who received prior training compared to those who did not receive prior training (F (1, 104) = .01, *p* = .92) (Figure 5).

Further assessment and comparison of virtual and in-person training knowledge gain, and demographic characteristics of participants will be conducted.

## Discussion

The training is the first-time virtual format has been used to train FCHVs in the country. This programme has shown early success in its deployment, leveraging local partners and in improving knowledge. Partnership with the local government was important to be able to secure a government building with projectors and adequate space for the in-person training. Internet lag was seen in the first virtual training during videos display and live interaction but was resolved by the second training. Having a local NGO partner that has a history of doing virtual seminars allowed them to fix this issue in a timely manner. A major challenge seen with the FCHV training is the limited time they must participate in the training programme due to responsibilities at home, and the need to travel long distances to come to training site (most times on foot). A strict start and end time was necessary to get through the training in timely fashion.

There is a need to equip FCHVs with the most appropriate information for Nepal to fight against the COVID 19 pandemic, as well as long-term challenges faced in post-COVID, especially in mental health. This pilot rapid training programme represents a feasible and locally appropriate approach for these low resource communities with simplified, and highly evidence-based information delivered in local languages. Collaborative partnerships between local grassroots organisations and government bodies have proved successful in getting this rapid training programme started. In a country with rugged roads, limited transportation access but with wide telecommunication access, such virtual training programmes can serve as examples for future successful response programmes as well. This work can serve as a model moving forward.

## Paper context

Nepal’s Female Community Health Volunteers (FCHVs) lacked appropriate training in key COVID 19 related topics which limited their involvement in pandemic response locally. This communication details the success of an innovative virtual training programme conducted by a community-based organisation in partnership with local government to train rural FCHVs. This work shows that public health programmers and local government can utilise virtual training programmes to rapidly equip local community health volunteers to improve their knowledge.
